# Desmoglein-2-Integrin Beta-8 Interaction Regulates Actin Assembly in Endothelial Cells: Deregulation in Systemic Sclerosis

**DOI:** 10.1371/journal.pone.0068117

**Published:** 2013-07-11

**Authors:** Betti Giusti, Francesca Margheri, Luciana Rossi, Ilaria Lapini, Alberto Magi, Simona Serratì, Anastasia Chillà, Anna Laurenzana, Lucia Magnelli, Lido Calorini, Francesca Bianchini, Gabriella Fibbi, Rosanna Abbate, Mario Del Rosso

**Affiliations:** 1 Department of Experimental and Clinical Medicine, University of Florence, Florence, Italy; 2 Department of Experimental and Clinical Biomedical Sciences, University of Florence, Florence, Italy; 3 Istituto Toscano Tumori, Florence, Italy; 4 National Cancer Research Centre “Giovanni Paolo II,” Department of Clinical and Neoplastic Experimental Oncology, Haematology Unit, Advanced Cellular Therapy Centre, Bari, Italy; China Medical University, Taiwan

## Abstract

**Background:**

The inability of endothelial cells of patients affected by the diffuse form of Systemic sclerosis (SSc) to perform angiogenesis is a marker of the disease. We previously demonstrated that desmoglein-2 reduction is a major difference between (SSc)-microvascular endothelial cells (MVECs) and normal (N)-MVECs. Here we investigated the role of desmoglein-2 in human N-MVECs and SSc-MVECs angiogenesis.

**Methodology/principal findings:**

Angiogenesis was studied by Matrigel invasion, capillary morphogenesis *in vitro* and Matrigel plug assay *in vivo*. Gene profiling was studied by Affymetrix technology and signal transduction by Western blotting. Colocalization was validated by immunoprecipitation and confocal microscopy. SiRNAs were used to validate the roles of specific molecules. We observed that desmoglein-2 co-localizes with integrin-beta8 in N-MVECs. This complex is required to signal through Rac, FAK, SMAD1/5 and MAP-kinases, promoting an angiogenic program. Inhibition of desmoglein-2 by *DSG2-*siRNA impaired actin stress fibres formation, capillary morphogenesis *in vitro* and angiogenesis *in vivo*. Transcriptome profiling after *DSG2* inhibition revealed alterations of several genes involved in actin organization. siRNA inhibition of integrin-beta8 and RAC2 also resulted into capillary morphogenesis impairment in N-MVECs, due to reduced expression of the same actin-assembly genes that were down-regulated by *DSG2* silencing. SSc-MVECs showed down-regulation of the same genes in *DSG2*-siRNA treated N-MVECs, suggesting that impairment of desmoglein-2/integrin-beta8 complex contributes to angiogenesis derangement in SSc. Transfection of *DSG2* in SSc-MVEC partially restored their angiogenic properties *in vitro*.

**Conclusions/significance:**

We have shown that impairment of actin assembly as a result of desmoglein-2/integrin-beta8 complex formation is a major factor contributing to angiogenesis deregulation in Systemic sclerosis.

## Introduction

We have previously studied Systemic sclerosis-microvascular endothelial cells (SSc-MVECs) as a model system to identify alterations accounting for anti-angiogenesis [1]–[4]. We observed that SSc-MVECs, which over-express pro-angiogenic factors, also over-produce anti-angiogenic molecules and lack operative systems required to perform angiogenesis. Reduced expression of desmoglein-2 (*DSG2*) in SSc-MVECs was one of the most striking differences, as demonstrated by differential transcriptome profiling and by immunohistochemistry of endothelial cells (EC) performed in patients affected by the diffuse form of SSc [3]. Although desmoglein-1/2 has been identified as a structural component of EC [5], [6], and has been described in many transcriptome-profiling studies of EC [7]–[9], its function in EC and in particular in angiogenesis has not yet been studied. Desmoglein-2 belongs to the family of desmosomal cadherins, involved in many biological processes including cell adhesion, morphogenesis, cytoskeletal organization and cell sorting/migration, as well as in pathological conditions such as cancer [10], [11]. The most studied cadherin of EC is vascular endothelial (VE)-cadherin (*CDH5*) which plays an important role in vasculogenesis and vascular remodelling [12], although its absence still allows EC to assemble to form vascular networks, indicating that VE-cadherin is not essential in this process [13]. Small GTPases of the Rho family regulate cadherin-based cell adhesion and linkage of cadherins to the actin cytoskeleton, which is responsible for strong adhesion [10]. The intracellular regions of desmosomal cadherins, such as desmoglein-2, interact with plakoglobin and with plakophilin, which in turn links to desmoplakin and consequently with the intermediate filament network. Plakophilin induces filopodia, reduces cell contacts and stimulates the formation of motility-associated structures. The association of plakophilin at the crossroads of adhesion and motility points to an important role of desmoglein in migration, wound healing and tissue formation through its possibility to regulate actin dynamics [14]. Since ECs contain a large number of filopodia, which sense gradients of guidance cues and consequently turn the cell towards chemoattractants and angiogenic factors, we have studied by siRNA-dependent loss of function the role of desmoglein-2 in actin filament assembly in N-MVECs.

To identify the biological processes and pathways involved in the anti-angiogenic phenotype due to the reduced expression of desmoglein-2, we performed gene expression profiling of N-MVECs before and after siRNA silencing of the *DSG2* gene and investigated the alterations at the mRNA, protein and functional levels. Further, we have induced a desmoglein-2 gain-of-function in SSc-MVECs, observing a substantial increase in their angiogenic capabilities.

## Materials and Methods

### Ethics Statement

For animal studies the local Institutional Animal Care and Use Committee of the Medicine Faculty of Florence (Ospedale di Careggi) and the Italian Ministry of Health (Ministerial Decree n 21/2010, released on January 28, 2010) approved the experimental protocols described in the study. All surgery was performed under sodium pentobarbital anesthesia, and all efforts were made to minimize suffering. For human skin biopsies the local Ethical Committee of the Medicine Faculty of Florence approved the study protocols and participants provided their written informed consent to participate in this study.

### Subjects, tissue biopsies, endothelial cells

MVECs were isolated from 3 normal subjects (N-MVEC) and 3 SSc patients (SSc-MVEC) affected by the diffuse form of the disease. For patient selection and EC isolation refer to references 1–3. Where present, cell colonies were detached with EDTA, CD31-positive cells were subjected to immuno-magnetic isolation with Dynabeads-CD31 (Dynal Biotech) and characterized as described [1]–[3], [15]. Cells were maintained in complete EC-growth medium (ECGM) [1] and used between the 3^rd^ and 7^th^ passage in culture. Both N-MVECs and SSc-MVECS were screened for endothelial markers (CD31, KDR, CD105, vWF and ULEX lectin) at regular intervals, showing that the EC markers profile did not significantly change from the 3^rd^ to the 10^th^ passage.

### si-RNA treatment of N-MVECs

Targeting and not-targeting siRNAs were obtained from Dharmacon. Specific silencing of selected genes (*DSG2, ITGB8, RAC2*) was performed by transfection of N-MVECs with small-interfering-RNA (siRNA) (SMART-siRNA-pool, each pool targeting a single gene: human *DSG2, ITGB8, RAC2*, respectively), according to the manufactures's instruction. Not-targeting si-RNA pool constructs were used as negative control (siCONTROL). To validate gene silencing, the relevant mRNA levels were determined by a quantitative real-time (RT)–PCR, as described in the dedicated paragraph. To favour internalization into N-MVECs, siRNAs were incorporated into cationic liposomes, utilizing DharmaFECT transfection reagent. Cells were incubated with transfection mix (24–48 h for mRNA analysis and 48 h for protein and phenotypic analysis, respectively).

### RNA extraction and Microarray Analysis

Total RNA was isolated from N-MVECs and SSc-MVECs using RNeasy Kit (QIAGEN). Gene expression profiles of N-MVECs and si*DSG2*-N-MVECs were obtained by Affymetrix technology and Human Genome U133 Plus 2.0 GeneChip, that contain 54,675 probe-sets allowing evaluation of the expression of 47,000 transcripts and variants. In order to prepare RNA target for gene expression analysis, the GeneChip® 3'IVT Express Kit was used (http://www.affymetrix.com). After scanning, data files were checked for quality parameters. Microarray analysis was performed according to Affymetrix suggestions (GeneChip Expression Analysis: data analysis fundamentals in http://www.affymetrix.com) and our previous experience [3], [16]–[18].

### Statistical analysis and functional classification of microarray data

Data have been deposited in the National Center for Biotechnology Information (NCBI) Gene Expression Omnibus (GEO; http://www.ncbi.nlm.nih.gov/geo) and are accessible through GEO Series accession number GSE21547 (http://www.ncbi.nlm.nih.gov/geo/query/acc.cgi? acc = GSE21547). Image and expression data files were generated with Affymetrix MAS 5.0. Low level and statistical analysis was done using R 2.9. Microarray data were first processed in R environment (http://www.r-project.org) by Affy-package to identify present/absent probe-set, and then subjected to a normalization step according to the Micro Array Suite (MAS) method (www.affymetrix.com, www.bioconductor.org) [19]. In order to identify differentially expressed genes in si*DSG2*-N-MVECs with respect to N-MVECs, we applied a t-statistic variant approach. We used the significance analysis of microarrays (SAM) method [20] by Siggenes package (www.bioconductor.org) [21].

Functional classification of the differentially expressed genes was done using Database for Annotation, Visualization and Integrated Discovery (DAVID) (http://david.abcc.ncifcrf.gov/) [22]. DAVID analysis was performed including the Gene Ontology databases, three pathway databases (Biological Biochemical Image Database, KEGG PATHWAY and BIOCARTA) and three functional categories (SP_PIR_KEYWORDS, COG_ONTOLOGY and UP_SEQ_FEATURE). The differentially expressed genes were used for cluster analysis by a Ward hierarchical clustering algorithm separately to samples and genes. To cluster samples we used the matrix of the Pearson's correlation coefficient, while for genes we used the matrix of the Euclidean distance. The cluster analysis and the heat-map were both performed using the R statistical environment.

### Real-Time PCR

To validate silencing experiments and microarray data, we tested 12 genes by quantitative RT-PCR using TaqMan ABI-PRISM 7900 (Applied Biosystems): *DSG2* gene, 4 genes with increased expression and 7 genes with decreased expression at microarray experiments. We used 25 ng of cDNA for each sample. The “Delta-delta method” was used for comparing relative gene expression results (Applied Biosystems). Expression of target genes was normalized to *GAPDH* and displayed as fold-change or log_2_ fold change relative to control RNA used as the calibrator. In Table S1 the tested genes and their assay ID are reported.

### Immunoprecipitation, Western blotting and determination of RhoA and RAC-GTPase activity

For immunoprecipitation, 500 µg cell proteins from confluent MVEC monolayers were transferred into an Eppendorf microtube, the primary rabbit antibody (anti-ITGB8, Santa Cruz Biotechnology) was added in 0.1% BSA and incubated overnight at 4°C. To each lysate, protein A agarose beads (Sigma Aldrich) were added for 3 h at 4°C. Beads were collected by centrifugation and the supernatant was stocked for further Western blotting. Aliquots of the pellets were processed, electrophoresed and blotted as previously described [1], [3]. After incubation with blocking solution, membranes were probed with an anti-desmoglein-2 mouse monoclonal antibody (Chemicon International). After incubation with horseradish peroxidase-conjugated anti-mouse or anti-rabbit IgG (Amersham Biosciences), immune complexes were detected with the Amersham Biosciences ECL detection system. Membranes were exposed to autoradiographic films (Hyperfilm MP; Amersham Biosciences). After incubation with stripping solution, the membrane was washed, incubated with blocking solution and re-probed with anti-integrin antibodies. For other Western blotting, 40–100 µg of cell extract protein were electrophoresed in 12% SDS polyacrylamide gel under reducing conditions and blotted to a polyvinylidene difluoride membrane (Hybond-C Extra; Amersham Biosciences). The membrane was incubated with 5% skim milk in 20 mM Tris-buffer to block non-specific binding and probed with primary antibody to desmoflein-2 (1 mg/ml, 1∶500) (Chemicon International), phospho-ERK1/2 (p42/p44) (200 µg/ml,1∶500) (Cell Signaling Technology), ERK-2 (200 µg/ml, 1∶500), p38^MAPK^ (250 µg/ml,1∶200) (Chemicon International), phospho-p38^MAPK^ (250 µg/ml, 1∶500) (Biosource International), SMAD1/5 (100 µg/ml,1∶500), phospho-SMAD1/5 (Ser463/465) (100 µg/ml, 1∶500), phospho-FAK (tyr576/577) (100 µg/ml, 1∶500), FAK (200 µg/ml,1∶500) (Cell Signaling Technology). After incubation with horseradish peroxidase-conjugated donkey anti-mouse or anti-rabbit IgG (1∶5,000) (Amersham Bioscience), membranes were exposed to autoradiographic films (Hyperfilm MP). Protein-antibody complexes were revealed by an Odyssey Infrared Imaging System, using as fluorescent secondary antibodies IRDye 800 CW goat anti-mouse IgG (1∶12000) (LI-COR Biosciences). N-MVECs from different experimental conditions and SSc-MVECs were lysed in RIPA buffer, the lysates were clarified by centrifugation, and RhoA-GTP or RacGTP were quantified. Briefly, lysates were incubated with 10 μg Rhotekin-glutathione S-transferase (GST) fusion protein (Upstate) or p21 activated kinase-GST fusion protein, both absorbed on glutathione-Sepharose beads for 1 h at 4°C. Immunoreactive RhoA and Rac were quantified by Western blot.

### Invasion assay and proliferation

MVECs invasion through Matrigel-coated porous filters was evaluated by the Boyden chamber [1]. 8×10^3^ MVECs were placed in the upper chamber in culture medium containing 2% FCS. After 6h filters were removed and fixed in methanol. Non-migrated cells were mechanically removed from the upper surface and the migrated cells on the lower filter surface were stained and counted under a light microscope (40X) in 10 random fields per each well. Each point was performed in triplicate. Mean values of migrated cells for each point were calculated ± SD. Cell proliferation was evaluated by cell counting.

### Cell Viability Assay

The viability N-MVECs under each relevant condition was determined by a cell proliferation assay using WST-1 reagent (Roche). WST-1 is a water-soluble sulfonated tetrazolium salt that is cleaved by cellular succinate-dehydrogenases in living cells, yielding dark blue formazan. Damaged or dead cells exhibit reduced or no dehydrogenase activity. Briefly, N-MVECs were plated onto a 96-multiwell plate in quadruple and treated with siRNAs as described above. After 72 hours WST-1 solution/culture medium (5 mmol/l, 1∶9) was added to each well. Following 2-hours incubation at 37°C, absorbance at 450 nm (reference at 630 nm) was measured by a Multiskan JX microplate reader. Percentage of cell viability was calculated based on the absorbance measured relative to that of the untreated control cells maintained in culture medium alone.

### Matrigel-sponge-assay in mice

Aliquots of 50 µl of serum-free ECGM, containing 50 U/ml heparin and 600 ng/ml VEGF, with or without *DSG2*-siRNA, combined with DharmaFect, were added to un-polymerized Matrigel at 4°C at a final volume of 0.6 ml. Matrigel suspension was injected subcutaneously into flanks of C57/BL6 male mice (Charles River) using a cold syringe. At body temperature Matrigel polymerizes and becomes vascularized within 4 days in response to angiogenic substances. Groups of 4 pellets were injected for each treatment. Pellets were removed, photographed with a stereo-microscope, minced and diluted in water to measure hemoglobin content with a Drabkin reagent kit (Sigma Aldrich).

### In vitro capillary morphogenesis assay

Matrigel (0.5 ml; 10–12 mg/ml) was pipetted into 13-mm tissue culture wells and polymerised for 30 min to 1h at 37°C [4]. MVECs were plated (60×10^3^/ml), in complete MCDB medium, supplemented with 30% FCS, and 20 µg/ml EC-growth-supplement (ECGM). Morphogenesis was evaluated after 6 h with an inverted microscope (Leitz DM-IRB) equipped with a digital analysis system. Results were quantified at 6h by measuring the percent field occupancy of capillary projections. Six to nine photographic fields from three plates were scanned for each point. Results were expressed as percent field occupancy ± SD with respect to control fixed at 100%.

### Immunofluorescence and Confocal-Laser-Scanning-Microscopy (CLSM)

N-MVECs and SSc-MVECs were grown on coverslips in ECGM, fixed in paraformaldehyde and permeabilised according to routine immuno-cytochemistry methods. Nuclei were stained with DAPI (10 μg/ml) (Sigma) for 15 min at RT. The anti-human primary antibodies used were: anti-integrin-beta8 (Santa Cruz Biotechnology); anti-desmoglein-2 (Chemicon International). The secondary antibodies were Alexa 488-conjugated goat anti-mouse IgG (1∶200) (Molecular Probes), Texas Red-conjugated goat anti-mouse IgG (1∶100) (Chemicon International) and Texas Red-conjugated goat anti-rabbit IgG (1∶200) (Molecular Probes). TRITC-labeled phalloidin (Sigma) was applied to the cells to visualize the arrangement of actin cytoskeleton. The coverslips containing the immunolabeled cells were mounted with an anti-fade mounting medium (Biomeda) and observed under a Bio-Rad MRC 1024 ES CLSM. The cells were examined with a Nikon Plan Apo X60-oil immersion objective using an excitation wavelength appropriate for Alexa 488 (495 nm) and Texas Red (595 nm). A series of optical sections were then taken through the depth of the cells with a thickness of 1μm at intervals of 0.8 μm. Quantitative analysis and colocalization of antigens were performed on acquired images analyzed as individual channel. The Manders overlap coefficients (M1 and M2) were determined using JACoP plugin and also calculated using the intensity correlation analysis plugin of the open-source softwer WCIF- Image J 1.44 as previously described [23]. We used the same operating mode of image analysis: Manders overlap coefficients indicate an overlap of the signals and thus represent the degree of colocalization between the red and green pixels: their values range from 0 (no overlap) to 1 (complete overlap). The background signal on each image was initially corrected using the Image J background subtraction function and, whenever possible, single cells on the images were selected using the Iasso tool (which defines a so-called “region of interest”). Colocalization was then calculated, after choosing the threshold values for the green and red channels, with the aforementioned plugin on the regions of interest previously defined. Quantization of Desmoglein-2 fluorescence and Integrin-beta8 fluorescence was conducted on z stacks of 70 sections corresponding to a thickness of approximately 0.8 μm passing through the middle of the cells. Regions of interest were manually drawn around each cell, and their integrated intensity after background correction was measured in Desmoglein-2 and Integrin-beta 8 channels. Image J software was used for fluorescence quantization, and Origin 6.1, Version 6.1052 (B232) was used for statistical analysis. Results are expressed as mean ± SD. Multiple comparisons were performed by 1-way ANOVA with Bonferroni correction. A *P* value <0.05 was considered statistically significant. Some immunofluorescence experiments with TRITC-labeled phalloidin, aimed to show the involvement of specific signalling pathways in actin polymerization, were performed in the presence of the following signalling inhibitors: FAK inhibitor 14 (Santa Cruz Biotech), MEK inhibitor UO126 (Promega), p38 inhibitor SB202190 and TGFβ receptor type I and type II inhibitor LY2109761 (both from SelleckBio.com).

### Transient transfection of SSc-MVECs with DSG2

For transient transfection of the human *DSG2* gene, SSc-MVECs (6×10^6^ cells) were incubated in 500 µl of serum-free OPTI-MEM (Life Technologies) with the addition of recombinant vector (CMV6-XL DSG2) or pCMV6-XL alone as empty vector control (15 µg each) (OriGene Technologies) and then were electrophorated using a Bio-Rad Gene Pulser apparatus.

### Statistical analysis

Results are expressed as mean ± SD. Comparisons were performed by the Student test, differences were considered statistically significant at p<0.05.

## Results

### DSG2 silencing in N-MVECs and its effects on in-vitro parameters of angiogenesis and in vivo Matrigel sponge assay

**Figure 1:**
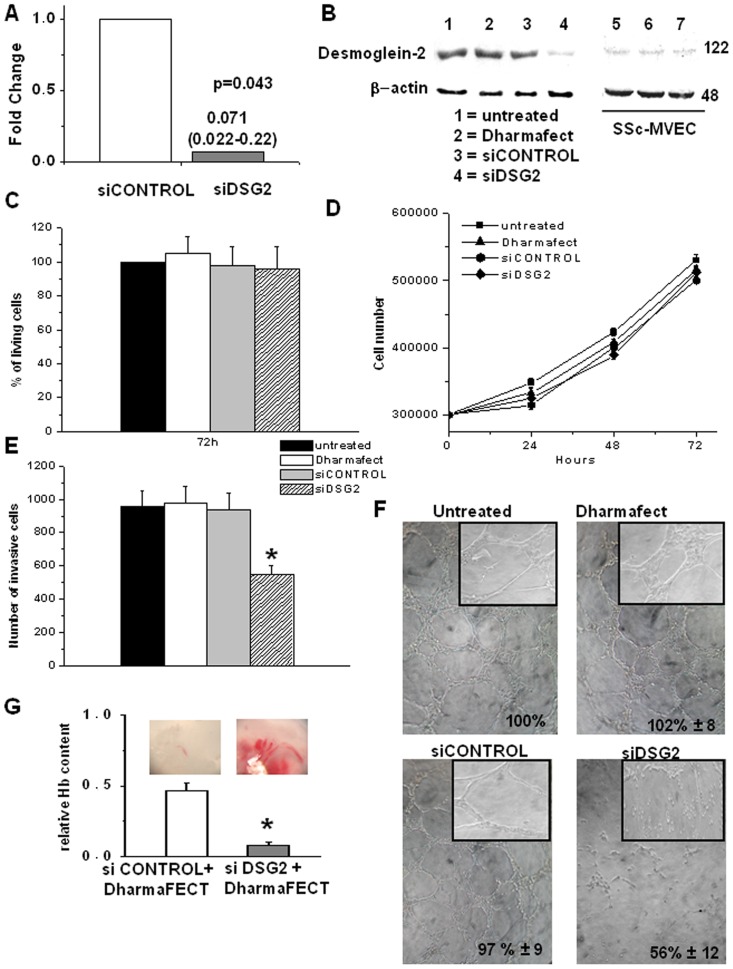
Effects of *DSG2* silencing on *in vitro* parameters of angiogenesis of N-MVECs and *in vivo* Matrigel sponge assay. A) *DSG2* RT-PCR after *DSG2* silencing. Data are expressed as fold change (siCONTROL-N-MVEC = 1). B) Representative desmoglein-2 Western blotting after *DSG2* silencing. Results shown are representative of similar data obtained in 3 different experiments; molecular weights markers are to the right; beta-actin: loading control. The blot on the right refers to desmoglein-2 expression in three different SSc-MVEC lines (5, 6, and 7) used in the present study. C) Cell viability evaluated by WST-1 assay, D) proliferation and E) matrigel invasion in *DSG2* silenced and control N-MVECs. Results are the mean ± SD of three different experiments performed in triplicate. *p<0.05, significantly different from control. F) Capillary morphogenesis at 6 h after seeding in Matrigel of control and treated N-MVECs. Numbers: percent field occupancy, taking control as 100%. Insets: morphology 24 hours after seeding. Data are from 3 experiments performed in triplicate. G) si*DSG2*-inhibition of angiogenesis in the Matrigel sponge model in mice. Upper part: angiogenesis quantification by haemoglobin content of each sponge. Pictures: ZEISS SR stereomicroscope aspect of the sponges under conditions corresponding to the histograms. Graphs are mean ± SD; * p<0.05. Results are the mean of three experiments (one animal for each condition, four Matrigel sponges in each animal).

After silencing of *DSG2* in N-MECs (si*DSG2*-N-MVECs), *DSG2* expression levels were reduced about 14-fold with respect to control cells (figure 1A). This correlated with a decrease in desmoglein-2 protein expression, as determined by Western blotting (figure 1B), following *DSG2*-siRNA treatment, showing *DSG2* down-regulation similar to levels expressed by SSc-MVECs (shown in lanes 1, 2 and 3 on the right). While cell viability (figure 1C) and cell proliferation (figure 1D) of N-MVECs resulted unaffected by siRNA treatment, Matrigel invasion (figure 1E) and capillary morphogenesis (figure 1F) were impaired. These results are similar to those previously reported for SS-MVECs [1].

Vascularization was evaluated in injected Matrigel sponges. Matrigel, containing serum-free ECGM with heparin and VEGF, with or without *DSG2*-siRNA, combined with DharmaFECT, was injected subcutaneously into the flanks of C57/BL6 male mice. *DSG2*-siRNA inhibited Matrigel vascularization after 4 days (figure 1G), in agreement with the effects observed *in vitro*.

### Affymetrix Gene Expression Profiling

To investigate the biological processes and pathways altered by reduced expression of *DSG2*, Affymetrix gene expression profiling of N-MVECs before and after siRNA silencing of *DSG2* was performed. Throughout all gene expression profiling experiments N-MVECs 1, 2 and 3 were used at passages 4, 4 and 5, respectively in order to minimize changes of gene expression related to cell passaging.

After data processing and application of the filtering criteria, the average of analyzable probe sets numbered 20,007 (37% of the 54,675 probe-sets represented in the GeneChip). By using the significance analysis of microarrays (SAM), we observed 2,945 transcripts differentially expressed in si*DSG2*-N-MVECs with respect to N-MVECs (GEO Series accession number GSE21547: supplementary file GSE21547_TableDE.xls.gz: http://www.ncbi.nlm.nih.gov/geo/query/acc.cgi?token=hbwpnommaskiexe&acc=GSE21547) Two hundred and twenty were expressed sequence tags (ESTs) not associated with a known gene (GEO Series accession number GSE21547: supplementary file GSE21547_TableDE.xls.gz: http://www.ncbi.nlm.nih.gov/geo/query/acc.cgi?token=hbwpnommaskiexe&acc=GSE21547)One thousand and seven hundred and two genes showed an increased expression and 1,243 genes had a reduced expression in the si*DSG2*-N-MVECs compared with N-MVECs (GEO Series accession number GSE21547: supplementary file GSE21547_Table DE.xls.gz: http://www.ncbi.nlm.nih.gov/geo/query/acc.cgi?token=hbwpnommaskiexe&acc=GSE21547)

### Classification of differentially expressed genes and cluster analysis

We performed the functional classification of the 2,945 differentially expressed genes using DAVID analysis and including in the analysis information from the Gene Ontology database (Molecular Function, Biological Process and Cellular Component), three pathway databases (Biological Biochemical Image Database, KEGG PATHWAY and BIOCARTA) and three functional categories (SP_PIR_KEYWORDS, COG_ONTOLOGY and UP_SEQ_FEATURE). Several classes were significantly associated with si*DSG2*-N-MVECs. Among the over-represented annotation terms, we observed a high number of functional terms and pathways implied in cytoskeleton organization and biogenesis, angiogenesis, blood vessel development. These data, together with those of figure 1, suggested that cytoskeleton deregulation could be a crucial mechanism of the altered phenotype of si*DSG2*-N-MVECs. Table 1 shows a selection of differentially expressed genes involved in cytoskeleton biogenesis/organization and angiogenesis.

**Table 1:** Differential expression of selected genes in siDSG2-N-MVECs, involved in cytoskeleton biogenesis and organization and angiogenesis.

Gene name	Gene Symbol	Expression
Cytoskeleton biogenesis and organization		
A kinase (PRKA) anchor protein 12	AKAP12*	↓
**actin related protein 2/3 complex, subunit 3, 21kDa**	**ARPC3**	↓
actin related protein 2/3 complex, subunit 5-like	ARPC5L	↓
capping protein (actin filament) muscle Z-line, beta	CAPZB	↓
catenin (cadherin-associated protein), beta 1, 88kDa	CTNNB1*	↓
catenin (cadherin-associated protein), delta 1	CTNND1*	↓
CDC42 effector protein (Rho GTPase binding) 2	CDC42EP2	↓
CDC42 effector protein (Rho GTPase binding) 3	CDC42EP3*	↓
cytoskeleton associated protein 5	CKAP5	↓
**diaphanous homolog 1 (Drosophila)**	**DIAPH1**	↓
**diaphanous homolog 2 (Drosophila)**	**DIAPH2**	↓
dynactin 6	DCTN6	↓
dynein, axonemal, heavy chain 5	DNAH5	↓
dynein, cytoplasmic 1, heavy chain 1	DYNC1H1	↓
dynein, cytoplasmic 1, intermediate chain 2	DYNC1I2	↓
dynein, cytoplasmic 1, light intermediate chain 2	DYNC1LI2	↓
**integrin, beta 8**	**ITGB8**	↓
**microtubule-actin crosslinking factor 1**	MACF1*	↓
mitogen-activated protein kinase kinase kinase 11	MAP3K11	↓
myosin IB	MYO1B*	↓
myosin ID	MYO1D	↓
neuronal cell adhesion molecule	NRCAM	↓
platelet-derived growth factor beta polypeptide (simian sarcoma viral (v-sis) oncogene homolog)	PDGFB*	↓
ras homolog gene family, member T2	RHOT2	↓
**ras-related C3 botulinum toxin substrate 2 (rho family, small GTP binding protein Rac2)**	**RAC2**	↓
rho/rac guanine nucleotide exchange factor (GEF) 18	ARHGEF18	↓
SMAD family member 1	SMAD1*	↓
syndecan 4	SDC4	↓
syndecan binding protein (syntenin)	SDCBP	↓
**thrombospondin 1**	THBS1*	↑
**Vinculin**	**VCL**	↑
**midline 1 (Opitz/BBB syndrome)**	MID1*	↑
**Angiogenesis**		
Interleukin 1, beta	IL1B	↓
matrix metallopeptidase 14 (membrane-inserted)	MMP14*	↓
laminin, alpha 4	LAMA4*	↓
Endothelin 1	EDN1	↓
**cadherin 5, type 2 (vascular endothelium)**	**CDH5**	↓
kinase insert domain receptor (a type III receptor tyrosine kinase)	KDR	↓
jagged 1 (Alagille syndrome)	JAG1*	↓
placental growth factor	PGF*	↓

The table summarizes selected genes that had a log2 expression greater than X and P values less than Y, chosen for their biological relevance in cytoskeleton assembly and angiogenesis.

Expression: up arrows  =  genes with increased expression in siDSG2-N-MVECs; down arrows  =  genes with decreased expression in siDSG2-N-MVECs. * =  genes resulted differentially expressed by more than 1 gene set. Genes chosen for real-time PCR validation or siRNA-dependent silencing are reported in bold characters.

The 2,945 differentially expressed genes were then used for cluster analysis (figure S1, dendrogram). The gene expression profiles clearly distinguished between si*DSG2*-N-MVECs and N-MVECs and the cluster analysis identified six groups of genes by similar expression levels (identified as box A-F in figure S1). Interestingly, in the cluster E, one of the gene cluster with the higher expression levels, we found many genes significantly associated with classification terms involved in cytoskeleton, cell motility and migration, and angiogenesis (table S2).

### Altered actin cytoskeleton assembly and Rho/Rac-GTPase activity in silenced N-MVECs

**Figure 2:**
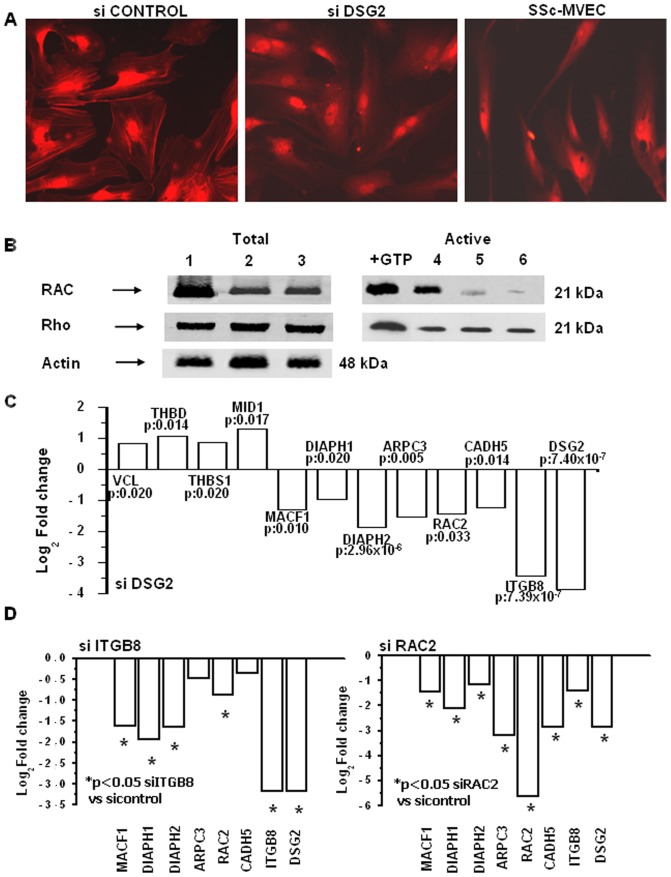
Effects of siDSG2 silencing in N-MVECs on stress fibres assembly and Rho/Rac transduction. A) Actin stress fibers revealed by labeled phalloidin with a Nikon Plan Apo X60-oil immersion objective. ImageJ 1.44 software was used for image acquisition. 94±4% of siCONTROL-treated N-MVECs showed actin-stress fibers organization, while 89±11% siDSG2-treated N-MVECs and 100% SSc-MVECs exhibited a complete absence of actin stress fibers. B) Rho/Rac activation. Lanes 1, 2 and 3: total Rac and Rho in N-MVECs treated with control non-targeting siRNA, anti-*DSG2* siRNA, and in SSc-MVECs, respectively; +GTP: positive control in N-MVECs lysate. Lanes 4, 5 and 6: constitutive Rho/Rac activation in control siRNA-N-MVECs, anti-*DSG2* siRNA-N-MVECs and in SSc-MVECs, respectively. Actin: loading control. Position of molecular weight markers in kDa is shown on the right. A representative result of a single N-MVEC and SSc-MVEC line is shown. Similar results were obtained with all the N-MVEC and SSc-MVEC lines used in this study. C) RT-PCR validation of selected genes following *DSG2* silencing in the three N-MVEC lines. D) Expression of the selected genes after *ITGB8* and *RAC2* silencing in N-MVECs. Results were normalized to GAPDH and fold changes of silenced-N-MVECs were calculated relative to the N-MVECs treated with control not-targeting siRNA. Data are expressed as log_2_ of fold change.

To verify whether *DSG2* silencing altered actin assembly, thus accounting for inhibition of angiogenesis *in vitro* and *in vivo*, we visualized actin organization in control and si*DSG2*-N-MVECs, as related to SSc-MVECs. Actin stress fiber disassembly was found in si*DSG2*-N-MVECs (figure 2A) and a similarly altered actin organization like that of SSc-MVECs. Since small GTPases of the Rho/Rac family mediate cadherin-dependent cytoskeletal rearrangements and cell motility [10], as well as integrin-mediated outside-in signals to actin cytoskeleton, we studied Rho/Rac activation by Western blotting. N-MVECs and SSc-MVECs both express Rac (figure 2B), whose activated form, evident in N-MVECs, resulted barely detectable in both si*DSG2*-N-MVECs and in SSc-MVECs. Interestingly, DSG2-silenced N-MVECs expressed lower amounts of Rac, in line with data obtained by microarray in si*DSG2*-N-MVECs, while no modification of Rho was observed (figure 2B).

### Real Time-PCR validation of microarray data

By real time-PCR, we assessed the expression of 12 selected differentially expressed genes: seven genes with a decreased expression (*MACF1*, which stabilizes actin at sites where microtubules and microfilaments meet; *DIAPH1*, *DIAPH2* and *ARPC3*, involved in regulation of actin polymerization; *CDH5*, whose intracellular partners α-catenin, β-catenin and vinculin interact with the actin network; the integrin *ITGB8*, which mediates cell-cell, cell-ECM interactions and cytoskeleton assembly in ECs; *RAC2*, involved in endothelial integrin signalling, and *DSG2*. Four genes with an increased expression (*VCL*, a cytoskeletal protein involved in anchoring F-actin to the membrane; *THBD*, a marker of endothelial injury; *THBS1*, an angiogenesis inhibitor; *MID1*, a microtubule-associated protein) were assessed in parallel (Figure 2C).

### Down regulation of the actin-cytoskeleton regulating genes by silencing of ITGB8 and of RAC2 in N-MVECs

We examined by real time-PCR the expression of *MACF1*, *DIAPH1*, *DIAPH2*, *ARPC3*, *RAC2*, *CDH5*, *ITGB8* and *DSG2* following siRNA-dependent silencing of either *ITGB8* or *RAC2* (Figure 2D). The data show that silencing of either gene provided a gene expression pattern similar to that obtained upon silencing of *DSG2* (shown in figure 2C). These results, coupled with those shown in figure 2B, were strongly suggestive of a possible functional and spatial relationship of the protein products of *DSG2*, *ITGB8* and *RAC2* genes.

### Colocalization of integrin-beta8 and desmoglein-2 in N-MVECs and related transduction pathways

**Figure 3:**
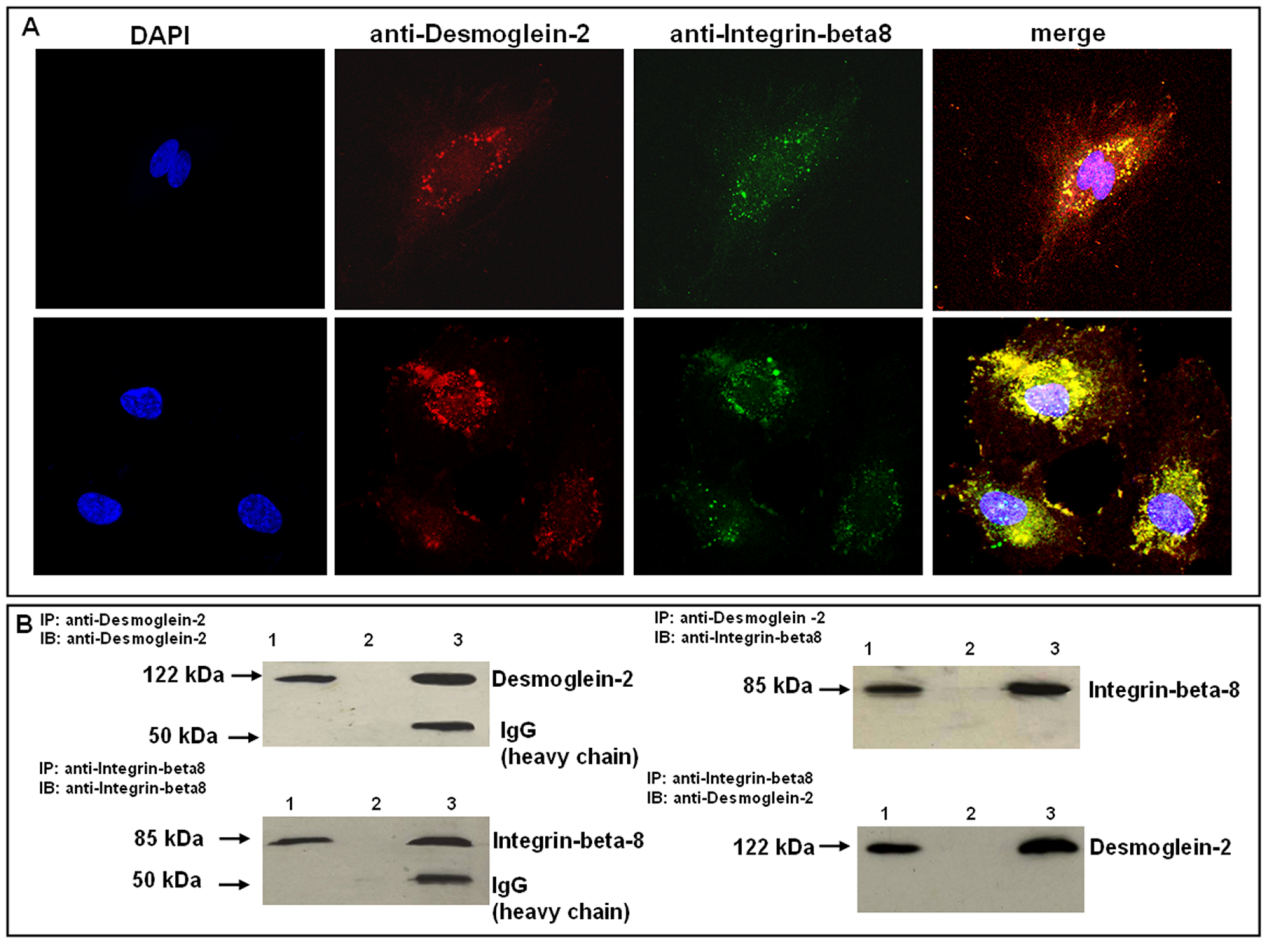
Colocalization of desmoglein-2 and integrin-beta8 in N-MVECs. A) Immunostaining of N-MVECs with anti-desmoglein-2 and anti-integrin-beta8 antibodies (nuclei stained with DAPI) in a single N-MVEC (upper row) and in a group of N-MVECs (lower row). The results shown are indicative of experiments performed on 3 different N-MVEC cell lines. Original magnification: ×60 (Bio-Rad MRC 1024 ES Confocal Laser Scanning Microscope). B) Co-immunoprecipitation of integrin-beta8 and desmoglein-2. The lanes 1, 2 and 3 of each blot represent 1) input: total lysate, 2) protein A beads alone, 3) protein A beads + anti-Desmoglein-2 or anti-Integrin-beta8 antibody, respectively. Left side: immuno-blotting with anti-desmoglein-2 (upper part) and anti-integrin-beta8 (lower part) antibodies of the immuno-precipitates obtained with the same antibodies. Right side: immuno-blotting with anti-integrin-beta8 antibodies of the immuno-precipitate obtained with anti-desmoglein-2 antibodies (upper part), and immuno-blotting with anti-desmoglein-2 antibodies of the immuno-precipitate obtained with anti-integrin-beta8 antibodies (lower part). Numbers on the left indicate molecular weight (kDa) of the revealed bands. IP: immuno-precipitate; IB: immuno-blotting. The data shown represent a typical experiment out of three experiments on each N-MVEC line, all of which gave similar results.

**Figure 4:**
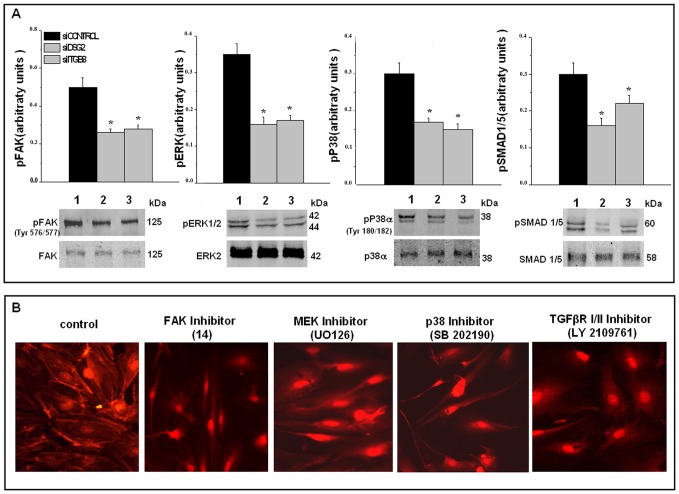
Integrin-dependent and integrin-independent transduction pathways in N-MVECs and their modulation by silencing of *DSG2* and *ITGβ8*. Panel A: Western blotting. In each Western blot lane 1 indicates control conditions (1), lane 2 the effect of si*DSG2* (2), and lane 3 the effect of si*ITGβ8* (3). Position of molecular weight markers in kDa is shown on the right. In each blot the non-phosphorylated form of the molecule is used as a loading control. The results shown have been selected among three different series of experiments that gave similar results in three different N-MVEC cell lines. For quantification, each electrophoresis pattern was subjected to densitometry. Histograms represent the average values ± SD of three different experiments performed in three different N-MVECs cell lines. *p<0.05, significantly different from control. Panel B: TRITC-labeled phalloidin immunofluorescence of stress fibers in control and in N-MVECs treated overnight with 5 µM FAK inhibitor 14, 30 µM MEK inhibitor UO126, 300 nM p38 inhibitor SB 202190, 10vµM TGFβRI/II inhibitor LY2109761.

Because in contrast to integrins and classical cadherins, desmogleins are not known to elicit intracellular signalling [24], and because our results indicated that *DSG2* and *ITGB8* silencing produced angiogenesis inhibition and a similar pattern of expression in genes regulating actin in N-MVECs, we evaluated whether integrin-beta8 and desmoglein-2 colocalize in MVECs, in order to verify whether integrin-beta8 was present as a transduction partner in a putative desmoglein-2/integrin-beta8 complex in MVECs. Confocal Laser Scanning Microscopy was used to co-localize integrin-beta8 and desmoglein-2 by specific antibodies. Both antibodies provided a diffuse micro and macro-dotted distribution, together with a slight membrane localization that, upon merging, revealed an almost complete co-localization (figure 3A). Co-localization was validated by immuno-precipitation experiments (figure 3B). Previous data had suggested that the integrin-beta8 cytoplasmic domain did not interact with the cytoskeleton and with cytoplasmic signalling pathways in an adhesion-promoting fashion [25]. However, it still appeared to be involved in late events in shaping cell morphology, such as process extension, indicating integrin-beta8 as a “divergent” signalling integrin [25]. Integrin-beta8 can signal through at least two different pathways: a classical FAK-mediated pathway and a TGFβ-mediated one. The latter pathway relies on the property of integrin-beta8 to release ECM-entrapped TGFβ in an activated form [26]. We therefore examined a series of transduction pathways related to classical integrin-mediated signalling and to TGFβ-mediated signalling. Both FAK phosphorylation and TGFβ-dependent SMAD1/5 phosphorylation decrease upon silencing of *DSG2* or *ITGB8* (figure 4A). The main components of down-stream transduction MAPK signalling pathway, ERK1/2 and p38α, which control gene expression, also show decreased phosphorylation upon *DSG2* or *ITGB8* silencing. The functional role of these signalling pathways in the regulation of actin assembly in MVECs was confirmed by the use of specific inhibitors of FAK, MEK, p38 and TGFβRI/II (figure 4).

### Biological consequences of the silencing of the 7 selected genes with reduced expression in siDSG2-N-MVECs

**Figure 5:**
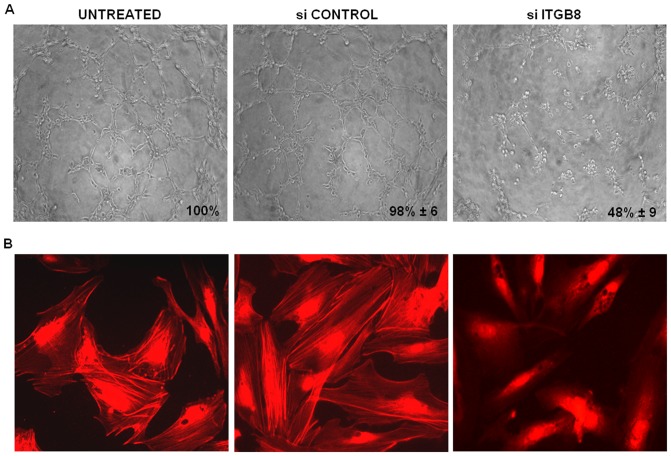
Effects of *ITGB8* silencing in N-MVECs. Panel A. Capillary morphogenesis following *ITGB8* silencing and relative controls. Pictures show the results of a typical experiment out of 3 experiments performed in triplicate with each N-MVEC line. Panel B: organization of actin stress fibers under the same conditions of A. Untreated N-MVECs showed actin stress fibers organization in 100% cells, siCONTROL-treated N-MVECs showed 93±6% of actin stress fibers-positive cells, while in *siITGB8*-treated N-MVECs actin stress fibers organization was absent in 91±8% cells.

In order to verify the role of the genes with reduced expression in si*DSG2*-N-MVECs, we performed the silencing of 7 selected genes: *MACF1*, *DIAPH1*, *DIAPH2*, *ARPC3*, *RAC2*, *CDH5*, and *ITGB8*, and evaluated the biological consequences of silencing in terms of capillary morphogenesis on Matrigel and stress fiber organization. ITGB8 silencing produced the inhibition of vessel-like structures and disrupted the actin cytoskeleton assembly, as shown by stress fiber disorganization with respect to untreated and control siRNA-treated MVECs (figures 5A and B). Silencing of the other genes gave similar results (data not shown).

### Evaluation in actin cytoskeleton regulating genes in SSc-MVECs and forced expression of DSG2 in SSc-MVECs

**Figure 6:**
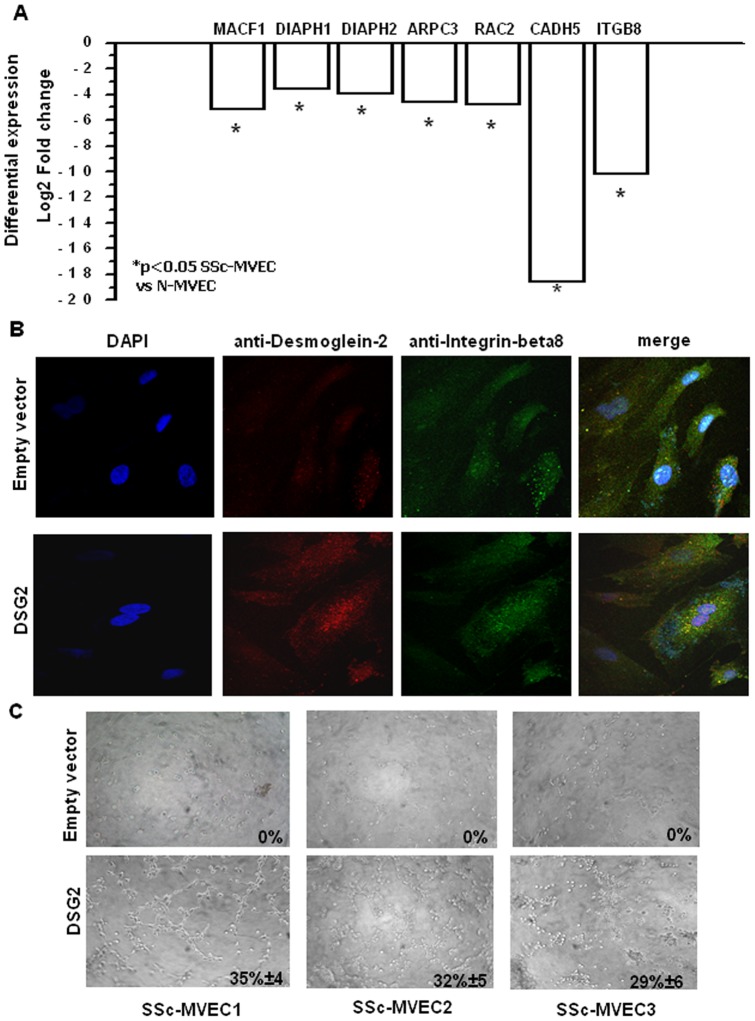
Real time PCR in SSc-MVECs of *MACF*, *DIAPH1*, *DIAPH*2, *ARPC3*, *RAC2*, *CDH5*, and *ITGB8*. Rescue of the angiogenic phenotype in SSc-MVECs by transient transfection. Panel A: RT-PCR of the relevant transcripts in SSc-MVECs, relative to N-MVECs. Data are expressed as log_2_ of fold change. Negative values indicate decreased expression levels. Panel B: Immunostaining of SSc-MVECs with anti-desmoglein-2 and anti-integrin-beta8 antibodies (nuclei stained with DAPI) after transient transfection with control vector (upper pictures) or *DSG2* transfection (lower pictures). The results shown are indicative of experiments performed on 3 SSc-MVEC cell lines. Original magnification: X60 (Bio-Rad MRC 1024 ES Confocal Laser Scanning Microscope). Panel C: Capillary morphogenesis in control and *DSG2*-transfected SSc-MVECs. Pictures show the results of a typical experiment out of 3 experiments performed in triplicate in each one of the different SSc-MVEC lines (SSc-MVEC 1, 2 and 3).

Our previous studies on transcriptome profiling of SSc-MVECs [3] tested 14,000 transcripts, as compared to the 47,000 transcripts of this study. The previous profiles included *DSG2* but did not assay the other genes involved in actin-cytoskeleton organization investigated and validated here. To investigate whether si*DSG2*-N-MVECs and SSc-MVECs shared alterations of the actin-cytoskeleton genes down-regulated by siDSG2, we assessed by RT-PCR the expression of *MACF1*, *DIAPH1*, *DIAPH2*, *ARPC3*, *RAC2*, *CDH5* and *ITGB8* genes in three SSc-MVEC lines (figure 6A). Our results showed that these genes had reduced expression in all the SSc-MVEC lines as compared with N-MVECs. To test the hypothesis that desmoglein-2 is required for efficient angiogenesis of N-MVECS that is lacing in SSc, we transiently transfected the three SSc-MVEC lines with *DSG2*. Immunofluorescence analysis of *DSG2*-transfected SSc-MVECs showed significantly increased levels of desmoglein-2 and integrin-beta 8 expression along with significantly increased desmoglein-2/integrin-beta 8 colocalization (figure 6B and table 2). Further, the *DSG*2 transfected SSc-MVECs partially recovered the capability to undergo capillary morphogenesis *in vitro* (figure 6C).

**Table 2:** Confocal microscopy analysis of SSc-MVECs transfected with *DSG2*.

	Mean desmoglein-2 relative intensity	Mean integrin-beta8 relative intensity	Colocalization (desmoglein-2 overlapping integrin- beta8)
Empty vector	142742±35997	242672±42488	0.629
*DSG2*	**+182%** *	**+210%** *	**0.793** *

* p<0.05, statistically significant.

## Discussion

We have demonstrated here that desmoglein-2 and integrin-beta8 are colocalized in N-MVECs and that the complex is required to deliver signals, mediated by the small GTPase Rac, by FAK and SMAD 1/5 phosphorylation, and by phosphorylation of the MAP-Kinase ERK 1/2 and p38α, which promote angiogenesis in N-MVECs. Inhibition of *DSG2* and/or *ITGB8* expression by siRNA impaired N-MVECs angiogenesis, inducing actin stress fibres disassembly. The effect depended on down-regulation of genes involved in actin assembly, such as *ARPC3*, *CDH5*, *DIAPH1*, *DIAPH2* and *MACF1*. Inhibition of each one of such genes produced actin disassembly and capillary morphogenesis impairment. The same molecules were also down-regulated in SSc-MVECs, suggesting that impairment of the desmoglein-2/integrin-beta8 complex contributes to angiogenesis derangement in the diffuse form of Systemic sclerosis.

Although desmoglein-1/2 has been identified as a structural component of EC intercellular junctions [5], and has been reported in transcriptome-profiling studies of ECs [3], [7]-[9], its role in the context of EC function has never been experimentally addressed. Our data identify unexpected properties of *DSG2* in regulation of actin dynamics in ECs.

Reducing *DSG2* expression with siRNA in N-MVECs to levels comparable with SSc-MVECs [3] produced a similar inhibition of angiogenesis *in vivo* and *in vitro*. The transcriptome-profiling of *DSG2-*silenced N-MVECs identified a number of differentially expressed genes associated with cytoskeleton organization, cell motility and migration, and angiogenesis, suggesting that cytoskeleton deregulation could be a crucial mechanism of the altered angiogenic phenotype of si*DSG2*-N-MVECs. In particular, in si*DSG2*-N-MVECs *ARPC3*, which promotes actin polymerization [27], had decreased expression suggesting a possible role in cytoskeleton disorganization. The protein encoded by the *MACF1* gene belongs to the plakin family of cytoskeletal linker proteins [28] and appears to stabilize actin at sites where microtubules and microfilaments meet. Actin polymerization also requires proteins known to interact with diaphanous protein *DIAPH1* and *DIAPH2*, that work through *RAC* activation [29]. Functioning as a classic cadherin, the protein encoded by *CDH5* gene plays an important role in EC biology through organization of the intercellular junctions and induction of actin assembly [10]–[13]. The *ITGB8* gene is a member of the integrin-beta family and encodes a single-pass type I membrane protein [30]. In general, integrin complexes mediate cell-cell and cell-extracellular-matrix interactions, as well as cytoskeleton organization [25]. The GTPase *RAC2* is involved in endothelial integrin signaling and in the postnatal neovascularization response *in vivo* [31]. Moreover, it has been shown that the small GTPase *RAC2* regulates actin structures in a variety of cells [32]. Down-regulation of this same set of genes was obtained by siRNA-dependent inhibition of *DSG2*, *ITGB8* or *RAC2*, suggesting that an outside-in informational flux dependent on their expression could be inhibited regardless of the silenced gene.

Because desmogleins are not known to elicit intracellular signalling [24], and *ITGB8* silencing resulted into angiogenesis inhibition and down-regulation of actin-regulating genes in a pattern similar to that elicited by *DSG2* silencing in N-MVECs, we hypothesized that integrin-beta8 was present as a transduction partner in a putative integrin-beta8/desmoglein-2 complex. This hypothesis was confirmed by co-localization of integrin-beta8 and desmoglein-2 in N-MVECs by Confocal Microscopy and immunoprecipitation. In particular, by confocal microscopy we have identified a non-conventional distribution of desmoglein-2, characterized by a diffuse micro and macro-dotted pattern over the cell surface in N-MVECs. This is not surprising, considering that desmosomes are not present in endothelial cells and that desmoglein-2 has been described as a novel solitary surface glycoprotein that is present in non-junction-restricted forms in cutaneous cells devoid of desmosomes, upon malignant transformation [33].

We also observed a complex array of signaling pathways that are likely to cooperate in integrin-beta8/desmoglein-2-dependent angiogenesis. Since integrin-beta8 is the signalling molecule of the integrin-beta8/desmoglein-2 complex, inhibition of signalling dependent on *DSG2* silencing is likely to be ascribable to a possible function of desmoglein-2 as an “adjuvant” molecule required for integrin-beta8 to exert its signalling function. The pathways involved range from FAK to the small GTPase RAC, the MAP-Kinase system, considered immediately upstream of gene expression, and, surprisingly, the TGFβ-dependent EC-restricted SMAD1/5 transduction pathway. An intimate relationship between some integrins and the activation of latent TGFβ1 has recently been described [26]. Integrin-dependent activation of latent TGFβ may disclose the full range of TGFβ activities, including angiogenesis promotion [34]–[36]. It is interesting to note that upon *DSG2* silencing in N-MVECs, down-regulation of Rac and of its activation is not counterbalanced by Rho activation. From a general point of view, motile cells assume several phenotypes, including a mesenchymal mode, in which the cells are elongated and fibroblast-like, and a distinct amoeboid mode, with less adherent properties and extensive membrane deformation. The so-called “mesenchymal-type movement”, which heavily depends on cell surface-associated proteases, has been shown to depend on activation of Rac and inhibition of Rho GTPases. In contrast the cell movement referred to as “amoeboid-like” has been associated with the loss of cell proteases activity and is characterized by an opposite phenotype of small GTPases [37], [38]. Therefore, a high Rho/Rac ratio identifies an amoeboid motility, while a low Rho/Rac ratio connotes a mesenchymal motility. However, the Rho/Rac balance always requires a proper functioning of the actin cytoskeleton, which is the most important pre-requisite in order to shift from a mesenchymal to an amoeboid movement style. Since the actin assembly genes are deregulated in the absence of desmoglein2/integrin-beta8 complex, si-*DSG2*-treated N-MVECs are unable to shift to an amoeboid movement, which may account for the absence of Rho activation.

Our results contribute to add new insights into the mechanisms of angiogenesis deregulation in Systemic sclerosis. In addition to *DSG2*, also *MACF1*, *DIAPH1*, *DIAPH2*, *ARPC3*, *RAC2*, *CDH5*, and *ITGB8* genes showed a reduced expression in SSc-MVECs, suggesting that the very poor expression of *DSG2* previously observed in our study of the differential gene expression profiling of N-MVECs and SSc-MVECs [3] may play a critical role in the pathogenesis of Systemic sclerosis angiogenesis impairment.

## Conclusions

Our data support a key role of the integrin-beta8/desmoglein-2 complex in the regulation of the angiogenic process. In agreement with the observations of this study, transient transfection of SSc-MVECs with *DSG2* restored the loss of co-localization between desmoglein-2 and integrin-beta8, allowing at the same time a partial rescue of their capillary morphogenesis ability *in vitro*. These data show that DSG2, a recently emerged component of ECs, is particularly important in the regulation of a correct angiogenesis process and that its loss plays a relevant role in the complex scenario of angiogenesis deregulation in Systemic sclerosis.

## Supporting Information

Figure S1 **Hierarchical cluster analysis of the 2,945 differentially expressed genes in N-MVEC subjected to DSG2 silencing with respect to N-MVEC.**
(TIF)

Table S1 **Gene Name, Gene Symbol and Assay ID (Applied Biosystems) of genes analyzed by real time PCR.**
(DOC)

Table S2 **Over represented terms after functional classification (DAVID analysis) of the genes found in the cluster E of Supplemental Figure S1, associated with cytoskeleton biogenesis and organization and angiogenesis.**
(DOC)
