# Correction: Desmoglein-2-Integrin Beta-8 Interaction Regulates Actin Assembly in Endothelial Cells: Deregulation in Systemic Sclerosis

**DOI:** 10.1371/annotation/b41766f2-c23d-455e-8d6e-e4bce5ae1d80

**Published:** 2013-07-26

**Authors:** Betti Giusti, Francesca Margheri, Luciana Rossi, Ilaria Lapini, Alberto Magi, Simona Serratì, Anastasia Chillà, Anna Laurenzana, Lucia Magnelli, Lido Calorini, Francesca Bianchini, Gabriella Fibbi, Rosanna Abbate, Mario Del Rosso

There was an error in the XML code causing the last author's first and last names to be mislabeled. Author Mario Del Rosso's correct surname is "Del Rosso" and correct first name is "Mario". The correct name for citation is "Del Rosso M". The expanded citation can be found below.

Desmoglein-2-integrin Beta-8 interaction regulates actin assembly in endothelial cells: deregulation in systemic sclerosis.

Giusti B, Margheri F, Rossi L, Lapini I, Magi A, Serratì S, Chillà A, Laurenzana A, Magnelli L, Calorini L, Bianchini F, Fibbi G, Abbate R, Del Rosso M.

PLoS One. 2013 Jul 11;8(7):e68117. doi: 10.1371/journal.pone.0068117. 

