# Supplementary figures and images for: Desmoglein-2-Integrin Beta-8 Interaction Regulates Actin Assembly in Endothelial Cells: Deregulation in Systemic Sclerosis
Source: PLoS One. 2013 Jul 11;8(7):e68117. doi: 10.1371/journal.pone.0068117 (PMC3708925; doi:10.1371/journal.pone.0068117)

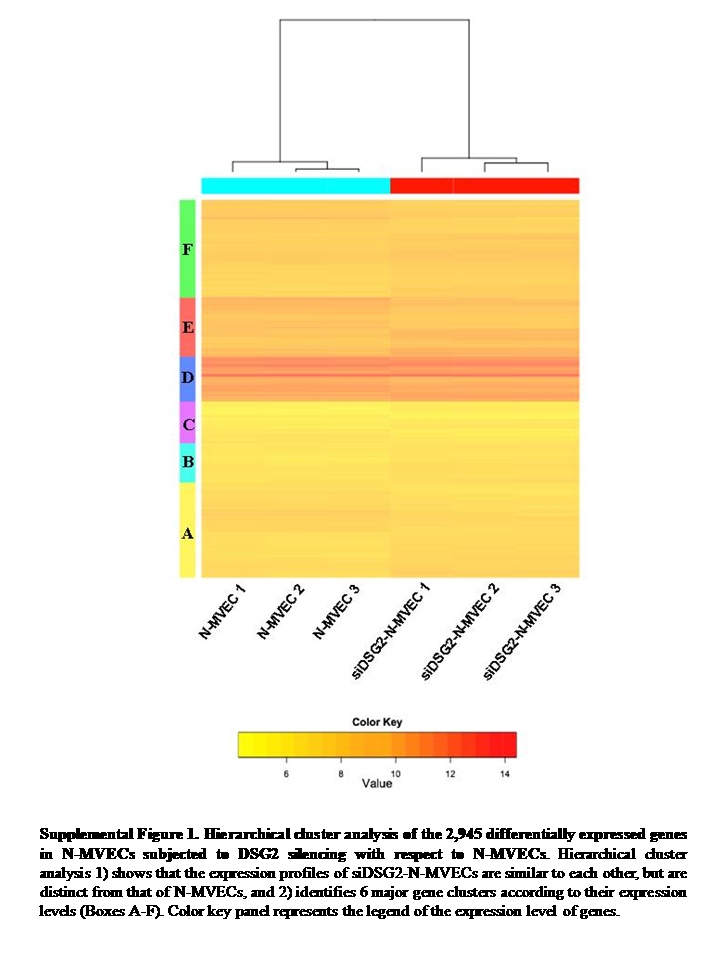

Supplement: Figure S1 — Hierarchical cluster analysis of the 2,945 differentially expressed genes in N-MVEC subjected to DSG2 silencing with respect to N-MVEC. (TIF) [file pone.0068117.s001.tif]
