# Supplementary material for: Desmoglein-2-Integrin Beta-8 Interaction Regulates Actin Assembly in Endothelial Cells: Deregulation in Systemic Sclerosis
Source: PLoS One. 2013 Jul 11;8(7):e68117. doi: 10.1371/journal.pone.0068117 (PMC3708925; doi:10.1371/journal.pone.0068117)
Supplement: Table S1 — Gene Name, Gene Symbol and Assay ID (Applied Biosystems) of genes analyzed by real time PCR. (DOC) [file pone.0068117.s002.doc]

**Table S1. Gene Name, Gene Symbol and Assay ID (Applied Biosystems) of genes analyzed by real time PCR.**

| **Gene Name** | **Gene Symbol** | **Assay ID**  **(Applied Biosystems)** |
| --- | --- | --- |
| Desmoglein 2 | DSG2 | Hs00170071_a1 |
| Vinculin | VCL | Hs00419715_m1 |
| Thrombomodulin | THBD | Hs00264920_s1 |
| Thrombospondin 1 | THBS1 | Hs00170236_a1 |
| Midline 1 (Opitz/BBB syndrome) | MID1 | Hs00166057_a1 |
| Microtubule-actin crosslinking factor 1 | MACF1 | Hs00201468_a1 |
| Diaphanous homolog 1 (Drosophila) | DIAPH1 | Hs00946556_a1 |
| Diaphanous homolog 2 (Drosophila) | DIAPH2 | Hs00246501_a1 |
| Actin related protein 2/3 complex, subunit 3, 21kDa | ARPC3 | Hs00855185_g1 |
| Ras-related C3 botulinum toxin substrate 2 (rho family, small GTP binding protein Rac2) | RAC2 | Hs01032884_a1 |
| Cadherin 5, type 2 (vascular endothelium) | CDH5 | Hs00901463_a1 |
| Integrin beta 8 | ITGB8 | Hs01110384_a1 |
| Glyceraldehyde-3-phosphate dehydrogenase | GAPDH | 4326317E |
