# Supplementary material for: Desmoglein-2-Integrin Beta-8 Interaction Regulates Actin Assembly in Endothelial Cells: Deregulation in Systemic Sclerosis
Source: PLoS One. 2013 Jul 11;8(7):e68117. doi: 10.1371/journal.pone.0068117 (PMC3708925; doi:10.1371/journal.pone.0068117)
Supplement: Table S2 — Over represented terms after functional classification (DAVID analysis) of the genes found in the cluster E of Supplemental Figure S1, associated with cytoskeleton biogenesis and organization and angiogenesis. (DOC) [file pone.0068117.s003.doc]

**Table S2. Over represented terms after functional classification (DAVID analysis) of the genes found in the cluster E of Supplemental Figure 1**, associated with cytoskeleton biogenesis and organization and angiogenesis

| Category | Term | p | Genes |
| --- | --- | --- | --- |
| Cytoskeleton biogenesis and organization | | | |
| SP_PIR_KEYWORDS | cytoskeleton | 4.51E-07 | JUB,LIMA1,UTRN,AKAP12,NEDD9,RAI14,FBLIM1,RDX,PDLIM1,DCTN6,CAPZB,GABARAP,VCL,TBCB,ARPC3,TBCA,ARPC2,FNBP1L,SDCBP,  SPTBN1MSN |
| GOTERM_CC_FAT | GO:0005856: cytoskeleton | 1.07E-04 | EIF6,JUB,DYNC1LI2,LIMA1,YWHAZ,C13ORF15SNCA,UTRN,AKAP12,NEDD9,C14ORF166,PDLIM1,RDX,NP,CAPZB,DSTN,VCL,STAU1,PSMB4,PTK2,FNTA,FRMD6,ARPC3,SULT1C4,ARPC2, MAP1LC3B,PPP2CB,MSN,DYNC1H1,TUBA1A,MAP1B,TANC1,RAI14,FBLIM1,DCTN6,GABARAP,CORO1C,TBCB,TBCA,FNBP1L,SPTBN1,SDCBP,PDCD6IP,SEPT7,DYNC1I2 |
| GOTERM_BP_FAT | GO:0008064: regulation of actin polymerization or depolymerization | 5.36E-04 | LIMA1,ARPC3,ARPC2,SPTBN1,RDX,CAPZB,DSTN |
| GOTERM_BP_FAT | GO:0030832: regulation of actin filament length | 6.30E-04 | LIMA1,ARPC3,ARPC2,SPTBN1,RDX,CAPZB,DSTN |
| GOTERM_BP_FAT | GO:0032956: regulation of actin cytoskeleton organization | 6.48E-04 | LIMA1,ARPC3,ARPC2,EDN1,SPTBN1,RDX,CAPZB,DSTN |
| GOTERM_BP_FAT | GO:0030834: regulation of actin filament depolymerization | 7.83E-04 | LIMA1,SPTBN1,RDX,CAPZB,DSTN |
| GOTERM_BP_FAT | GO:0032970: regulation of actin filament-based process | 7.83E-04 | LIMA1,ARPC3,ARPC2,EDN1,SPTBN1,RDX,CAPZB,DSTN |
| GOTERM_MF_FAT | GO:0008092: cytoskeletal protein binding | 9.35E-04 | GABARAPL2,PHACTR2,LIMA1,UTRN,SNCA,MAP1B,RDX,CAPZB,GABARAP,ANXA2P2,DSTN,VCL,CORO1C,FNTA,ARPC3,ARPC2,SPTBN1,SDCBP,MSN |
| GOTERM_BP_FAT | GO:0043244: regulation of protein complex disassembly | 0.0012 | LIMA1,MAP1B,SPTBN1,RDX,CAPZB,DSTN |
| GOTERM_BP_FAT | GO:0051493: regulation of cytoskeleton organization | 0.0016 | LIMA1,ARPC3,ARPC2,EDN1,MAP1B,SPTBN1,RDX,CAPZB,DSTN |
| GOTERM_CC_FAT | GO:0015629: actin cytoskeleton | 0.0017 | LIMA1,UTRN,SNCA,DCTN6,CAPZB,GABARAP,VCL,DSTN,CORO1C,ARPC3,ARPC2,SPTBN1,SEPT7,DYNC1I2 |
| SP_PIR_KEYWORDS | actin-binding | 0.0020 | CORO1C,PHACTR2,LIMA1,ARPC3,ARPC2,UTRN,SPTBN1,RDX,CAPZB,VCL,DSTN |
| GOTERM_CC_FAT | GO:0005875: microtubule associated complex | 0.0024 | DYNC1LI2,FNTA,MAP1B,DCTN6,DYNC1H1,GABARAP,DYNC1I2,STAU1 |
| GOTERM_CC_FAT | GO:0044430: cytoskeletal part | 0.0033 | EIF6,JUB,YWHAZ,LIMA1,DYNC1LI2,C13ORF15UTRN,NEDD9,C14ORF166,CAPZB,STAU1,DSTN,PSMB4,FNTA,ARPC3,MAP1LC3B,ARPC2,PPP2CB,TUBA1A,DYNC1H1,MAP1B,TANC1,DCTN6,GABARAP,TBCB,TBCA,SPTBN1,PDCD6IP,SEPT7,DYNC1I2 |
| GOTERM_BP_FAT | GO:0040012: regulation of locomotion | 0.0034 | JUB,NISCH,HIF1A,SNCA,EDN1,ITGA2,JAG1,ADAM9,KDR,VCL |
| PIR_SUPERFAMILY | PIRSF005455: microtubule-associated proteins 1 light chain 3A/3B | 0.0039 | GABARAPL2,MAP1LC3B,GABARAP |
| GOTERM_CC_FAT | GO:0015630: microtubule cytoskeleton | 0.0049 | JUB,DYNC1LI2,C13ORF15,MAP1B,NEDD9,C14ORF166,DCTN6,GABARAP,STAU1,PSMB4,FNTA,TBCB,TBCA,MAP1LC3B,PPP2CB,PDCD6IP,DYNC1H1,TUBA1A,SEPT7,DYNC1I2 |
| GOTERM_BP_FAT | GO:0006928: cell motion | 0.0051 | JUB,EDN1,ITGA2,CAPZB,DSTN,VCL,KDR,PTK2,HIF1A,ARPC3,CTGF,BTG1,ARPC2,SDCBPMSN,UBA52,FN1 |
| GOTERM_BP_FAT | GO:0032271: regulation of protein polymerization | 0.0051 | ARPC3,ARPC2,MAP1B,SPTBN1,RDX,CAPZB |
|  |  |  |  |
| GOTERM_BP_FAT | GO:0030334: regulation of cell migration | 0.0052 | JUB,NISCH,HIF1A,EDN1,ITGA2,JAG1,ADAM9,KDR,VCL |
| GOTERM_MF_FAT | GO:0003779: actin binding | 0.0057 | LIMA1,PHACTR2,UTRN,MAP1B,RDX,CAPZB,VCL,DSTN,CORO1C,ARPC3,ARPC2,SPTBN1,MSN |
| GOTERM_BP_FAT | GO:0030835: negative regulation of actin filament depolymerization | 0.0061 | LIMA1,SPTBN1,RDX,CAPZB |
| GOTERM_CC_FAT | GO:0030027: lamellipodium | 0.0080 | JUB,PTK2,ARPC3,NEDD9,RDX,CAPZB |
| SP_PIR_KEYWORDS | microtubule | 0.0093 | DYNC1LI2,TBCB,TBCA,MAP1LC3B,MAP1B,DYNC1H1,TUBA1A,GABARAPDYNC1I2 |
| SP_PIR_KEYWORDS | actin binding | 0.0095 | UTRN,SPTBN1,RDX,MSN |
| GOTERM_BP_FAT | GO:0051270: regulation of cell motion | 0.0109 | JUB,NISCH,HIF1A,EDN1,ITGA2,JAG1,ADAM9,KDR,VCL |
| GOTERM_BP_FAT | GO:0051494: negative regulation of cytoskeleton organization | 0.0115 | LIMA1,MAP1B,SPTBN1,RDX,CAPZB |
| GOTERM_BP_FAT | GO:0030833: regulation of actin filament polymerization | 0.0122 | ARPC3,ARPC2,SPTBN1,RDX,CAPZB |
| GOTERM_BP_FAT | GO:0007160: cell-matrix adhesion | 0.0136 | JUB,LIMS1,CTGF,ITGA2,ADAM9,FN1 |
| GOTERM_BP_FAT | GO:0030032: lamellipodium assembly | 0.0286 | JUB,CAPZB,VCL |
| GOTERM_BP_FAT | GO:0016477: cell migration | 0.0369 | JUB,PTK2,HIF1A,BTG1,CTGF,EDN1,SDCBP,MSN,KDR,FN1 |
| GOTERM_BP_FAT | GO:0051495: positive regulation of cytoskeleton organization | 0.0374 | ARPC2,EDN1,MAP1B,DSTN |
| GOTERM_BP_FAT | GO:0051693: actin filament capping | 0.0461 | SPTBN1,RDX,CAPZB |
| Angiogenesis | | | |
| GOTERM_BP_FAT | GO:0001525: angiogenesis | 6.98E-04 | PTK2,HIF1A,CTGF,EDN1,TGFBR2,LEPROT,JAG1,PLXND1,KDR,ANXA2P2 |
| GOTERM_BP_FAT | GO:0001568: blood vessel development | 0.0072 | PTK2,HIF1A,CTGF,EDN1,TGFBR2,LEPROT,JAG1,PLXND1,CDH5,KDR,ANXA2P2 |
| GOTERM_BP_FAT | GO:0048514: blood vessel morphogenesis | 0.0081 | PTK2,HIF1A,CTGF,EDN1,TGFBR2,LEPROT,JAG1,PLXND1,KDR,ANXA2P2 |
| GOTERM_BP_FAT | GO:0001944: vasculature development | 0.0086 | PTK2,HIF1A,CTGF,EDN1,TGFBR2,LEPROT,JAG1,PLXND1,CDH5,KDR,ANXA2P2 |
| GOTERM_BP_FAT | GO:0001569: patterning of blood vessels | 0.0424 | EDN1,TGFBR2,PLXND1 |
